# A Review of the Methods on Cobb Angle Measurements for Spinal Curvature

**DOI:** 10.3390/s22093258

**Published:** 2022-04-24

**Authors:** Chen Jin, Shengru Wang, Guodong Yang, En Li, Zize Liang

**Affiliations:** 1 The State Key Laboratory for Management and Control of Complex Systems, Institute of Automation, Chinese Academy of Sciences, Beijing 100190, China; jinchen2021@ia.ac.cn (C.J.); en.li@ia.ac.cn (E.L.); zize.liang@ia.ac.cn (Z.L.); 2 School of Artificial Intelligence, University of Chinese Academy of Sciences, Beijing 100049, China; 3 Peking Union Medical College Hospital, Beijing 100005, China; wangshengru72@pumch.cn

**Keywords:** scoliosis, Cobb angle measurement, deep learning, image enhancement

## Abstract

Scoliosis is a common disease of the spine and requires regular monitoring due to its progressive properties. A preferred indicator to assess scoliosis is by the Cobb angle, which is currently measured either manually by the relevant medical staff or semi-automatically, aided by a computer. These methods are not only labor-intensive but also vary in precision by the inter-observer and intra-observer. Therefore, a reliable and convenient method is urgently needed. With the development of computer vision and deep learning, it is possible to automatically calculate the Cobb angles by processing X-ray or CT/MR/US images. In this paper, the research progress of Cobb angle measurement in recent years is reviewed from the perspectives of computer vision and deep learning. By comparing the measurement effects of typical methods, their advantages and disadvantages are analyzed. Finally, the key issues and their development trends are also discussed.

## 1. Introduction

Spinal deformity refers to the appearance of morphological abnormalities in the coronal, sagittal, or axial position of the spine that deviate from the normal position. In 2014, Li, Y.W. et al. [1] screened 15,000 children who were randomly selected in the Luohe area of Henan Province, China, for spinal deformities and found that nearly two out of every 1000 children in the area suffered from congenital spinal deformities. The incidence of acquired spinal deformities is even more alarming. In the case of scoliosis, for example, epidemiological surveys show that more than 2% of adults in China and the United States suffer from scoliosis, and most of the onset occurs after adolescence [2,3].

Adolescent idiopathic scoliosis (AIS) is mostly mild and does not cause major health problems. However, there is a 10–20 percent chance that minor scoliosis will worsen further [4], while severe scoliosis can affect breathing [5]. Due to the progressive properties of scoliosis and the significantly higher probability of postoperative complications in adult patients than in adolescent patients, medical imaging diagnosis and screening for scoliosis are important [6].

**Figure 1:**
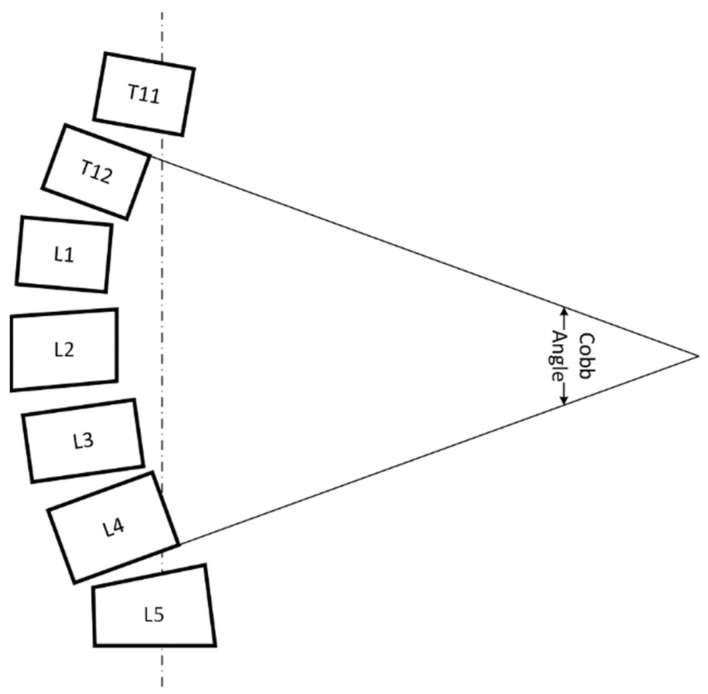
Schematic of Cobb angle measurements: the Cobb angle is defined as the angle between the extension line of the upper end plate of the most inclined vertebral body in a curved segment and the extension line of the lower end plate of the most inclined vertebral body below.

In order to scientifically assess the progression of the disease, a quantitative analysis of scoliosis is a necessary step in scoliosis treatment. Compared to other methods of assessing spinal curvature, the Cobb angle method is preferred for its better reproducibility [7], easier application, and suitability for measuring more severe spinal curvature. In 1966, the Scoliosis Research Society (SRS) adopted the Cobb angle as a standard method for quantifying scoliosis deformities. It remains the most commonly used method for assessing the curvature of the spine to date. The schematic of the Cobb angle is shown in Figure 1.

## 2. The Challenges of Cobb Angle Measurement

Clinically, a more than 5° change in Cobb angle can represent the progression of the disease course, which, in turn, affects the formulation of the doctor’s diagnosis and treatment plan. However, Jeffries, B.F. et al. [8] noted that a 5° change in Cobb angle might be affected by measurements rather than by an improvement or exacerbation of the course of the disease. Yang, W.Z. [9]’s research supported this opinion by pointing out the high inter- and intra-operator variability during the Cobb angle measurement. The sources of errors may be equipment errors, image errors, and subjective errors in the operator’s selection of the vertebral end. Therefore, computer-aided methods are considered superior in terms of reliability [10,11,12,13] because they can remove subjective errors. There are some computer-aided methods that measure spinal curvature without the help of traditional medical imaging (X-ray, CT, MRI, etc.), including body surface image screening [14,15] and motor system examination [16]. However, these methods either only allow for early screening or are too expensive to monitor the condition regularly. Therefore, traditional medical imaging is still an indispensable auxiliary tool in scoliosis treatment. However, scoliosis measurement software, which has a high market share at present, such as Surgimap [17], uses semi-automatic measurement methods. The operator needs to manually select the upper and lower ends of the vertebral body. Clearly, there is a huge market gap for commercial software for fully automatic measurement of Cobb angles using traditional medical imaging.

With the increasing demand for intelligent medical devices, automatic Cobb angle measurement is expected to assist in solving more complex problems in realistic medical scenarios and, therefore, faces more challenges. For example, during surgery, fully automated Cobb measurements can be used to assist with bone fixation. However, these methods suffer from parallax problems due to the narrow field of view for imaging equipment in the operating room. Additionally, in the regular Cobb angle review of the postoperative population, metal implants are prone to metal artifacts in 3D images and affect the robustness of the measurement method.

The rest of this paper is organized as follows. Several typical medical situations using automatic Cobb angle measurement and the preprocessing methods designed for them will be introduced in Section 3. In Section 4 and Section 5, we will introduce several automatic measurement methods developed for 2D and 3D images. In the last two sections, we will discuss the shortcomings of the current methods and future directions for the fully automated measurement of Cobb angles.

## 3. Typical Application Scenarios & Preprocessing Methods

Many factors can affect the imaging quality of medical images, so we selected several typical scenarios and summarized three preprocessing methods. These are considered to be effective in improving the robustness of automatic algorithms.

### 3.1. PDE Filters Used for Feature Enhancement

In X-ray images of the spine, the edges of the vertebral body are often degenerated and difficult to discern. The partial differential equation filter identifies the edges by calculating the gradient of the image (the area with a large gradient is most likely to be the edge) and smooths the image of the area, except the edge, hoping to strengthen the weak edge of the vertebral body and suppress image noise. Osher and Rudin et al. [18] proposed an iterative PDE to suppress noise. However, the inflection points of noise may be mistaken for edges. In response to this problem, Alvarez and Mazorra [19] proposed a shock-diffusion model with an anisotropic diffusion term. The proposed method provided a sharp cutoff at the edge, while the image was regularized. However, with a small standard deviation, the smoothing of the method will be insufficient. To solve the above problem, Gilboa et al. [20,21] improved their diffusion function to propose a complex diffusion partial differential equation filter based on the standard impact diffusion model.

Duong L et al. [22] compared these PDE models in terms of their noise reduction effects on spine images. The standard PDE model enhanced the noise while enhancing the vertebral body. Images processed by anisotropic diffusion PDE models were somewhat degenerate at the edges of the vertebral body. The complex diffusion PDE model performed best, not only effectively enhancing the vertebral body to eliminate noise but also minimizing the degeneration of the vertebral body edges.

Duong, L.et al. [22] also compared the classification accuracy of the SVM classifier before and after using the PDE filter. It was found that the accuracy of classifying the original image was between 50.2% and 84.0% without denoising, and the classification accuracy increased to 65.6–90.7% after using the PDE filter.

### 3.2. Preprocessing Methods Used to Deal with Parallax

Automated measurement of the Cobb angle can be used during surgery to confirm the restoration of the functional alignment of the spine. The X-ray machine used specifically for surgery usually has a narrow field of view [23], and the image of the spine is longer than the machine’s field of view, so it is necessary to stitch multiple captured images to form a complete image of the spine during surgery. Yaniv, Z. [24] proposed a method of moving the C-arm parallel to the front for image stitching to obtain panoramic images. However, he did not address the parallax problem during image registration. Methods of stitching X-ray images can be divided into intensity-based registration and feature-based registration. Intensity-based registration relies on large overlapping areas, thus increasing the radiation dose received by patients and medical staff. In response to this, Wang, L. [25] proposed a moving C-arm shooting method that does not require overlapping, thus reducing the number of shots and reducing the amount of radiation. The feature-based registration method is limited by the fact that the feature intensities of different images taken with X-rays may be completely different, making the stitching difficult. Since the spine consists of multiple vertebral bodies that cannot be completely parallel to the calibration plane, parallax effects can cause measurement errors in the image. There are manifold methods [26], multi-view methods [27], and pure rotating camera methods [28] to eliminate parallax, but they all have certain drawbacks.

Medtronic’s O-arm mobile 3D Cone-Beam CT imagers exhibit high intra- and inter-observer agreement. [29]. However, these images were of poor quality in obese patients and at the cervicothoracic junction. The 2D long film function in Medtronic’s latest O-arm has shown better performance in splicing imaging [30].

### 3.3. Preprocessing Methods Used for Metal Artifacts Reduction (MAR)

The corrective treatment of scoliosis is sometimes assisted by metal implants. Metals tend to cause metal artifacts in 3D images. MRI is not commonly used for spinal curvature measurement (unless spinal cord injury is suspected), while US is less disturbed by metal implants, and metal occlusion can be avoided by multi-angle scanning. However, metallic implants are prone to high-amplitude hardening artifacts in CT [31]. Therefore, algorithms have been proposed to eliminate metal artifacts in CT images. Metal artifact removal is usually carried out by segmenting the area affected by the artifact in the sinogram and replacing it with synthetic values, such as interpolation [32,33] and iterative reconstruction [34,35]. The interpolation method has high parameter requirements and can easily lead to the blurring of structures near the implant. The iterative reconstruction method has high computational complexity. Some unsupervised deep learning algorithms, such as AND-net [36] and CycleGAN [37], were introduced. Although good results have been achieved, unsupervised algorithms do not always produce filling information that conforms to the anatomical structure and are prone to secondary artifacts. Additionally, small metal implants tend to disappear in high-rise networks [38]. In addition, the existing MAR algorithms are all data-driven, so the dataset is extremely critical. However, when limited by the actual situation, it is impossible to find datasets that can implement supervised algorithms. Therefore, some researchers have introduced metal masks to images without metal artifacts to generate metal artifact images, conduct supervised training, and achieve good results [39,40].

## 4. Evaluation Methods of Cobb Angle in 2D Images

Due to its controllable radiation dose and low cost [41], X-ray is still the main way to assess scoliosis progression, and so it is used as the main medical imaging method for Cobb angle measurement. There are two main categories for the current commonly used methods of computer-aided measurements, which are image-enhancement-based methods and machine-learning-based methods.

### 4.1. Image Enhancement Method for 2D Image

X-rays of the spine are characterized by large noise, blurry edges, and possible occlusion between structures. Therefore, the image enhancement method is committed to extracting the features we need through means such as filtering. The biggest challenge the researchers encountered was how to segment the vertebral body from the image background.

#### 4.1.1. Semi-Automatic Method

**Figure 2:**
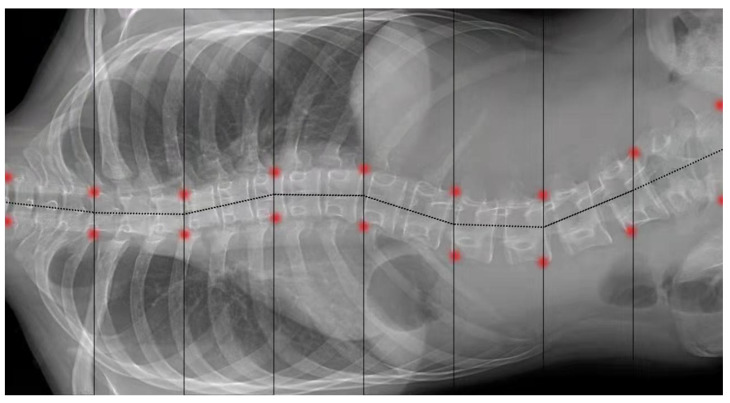
Schematic of octa segmentation method proposed by Chockalingam, N. et al. [42].

In earlier studies, because the vertebral body boundary is blurred and difficult to segment, researchers often use a semi-automatic method. Chockalingam, N. et al. [42] proposed a multi-segmented measurement using AP images. (Unless otherwise specified, all the 2D automatic measurement methods below use AP images). The program automatically divides the area of the spine (manually selected by the operator) into eight equal segments. The program automatically fits the midline of the spine according to the intersection of each parallel line and the spinal area that the operator manually identifies and then calculates the angle. An example diagram of its method is shown in Figure 2. Chockalingam, N. tested the intra-observer error and inter-observer error of the octa segmentation method and compared it with the manual method. The octave method has an excellent performance in the Technical Error of Measurement (TEM) and Average Reliability Coefficient (R). Table 1 shows the statistical measures for variability of the methods.

However, Chockalingam, N.’s method [42] can neither find the vertebral body with the largest tilt angle nor be sensitive in mild scoliosis scenarios. In order to measure the Cobb angle more accurately on more occasions, segmentation of the vertebral body is still unavoidable. Samuvel, B. et al. [43] proposed an automated Cobb angle measurement method for segmenting the vertebral body from the image using a mask. Yang, D. et al. [44] designed a two-stage, semi-automatic, ultrasound curve angle measurement method for adolescent idiopathic scoliosis. The method was composed of two processes: manual identification of spinal transverse processes and automatic angle measurement based on manually placed masks. The results showed that the proposed method had almost the same effect as the conventional method.

#### 4.1.2. Full-Automatic Method

Semi-automatic methods perform similarly to or better than manual methods. However, it is difficult for semi-automatic methods to remove subjectivity, which affects the measurement accuracy. There have been some works using image enhancement methods for the full-automatic measurement of Cobb angles. Some researchers calculate the Cobb angle by fitting the midline of the spine, while others measure it by extracting the upper and lower ends of the vertebral body. A very intuitive idea is to enhance the edges in the image and then abstract the irregular vertebral edges into quadrilateral shapes to separate the upper and lower ends of the vertebrae. Zhang, J. et al. [45] used Canny Operator to enhance the edges, making it easier for Hough transform to extract vertebral bodies from images. However, the Canny operator not only strengthens the edges of the vertebral body but also strengthens the edges of unrelated structures. Therefore, some researchers consider suppressing noise with filters. Anitha, H. et al. [46] proposed a method for image enhancement using three filters which are used for noise suppression, region of interest preservation, and edge extraction. This method automatically separates the vertebral ends and measures the Cobb angle. Comparing Anitha, H.’s method and manual measurement from the three dimensions of mean, standard deviation, and error, from the perspective of within and between observers, it was found that the former is better than the latter.

**Figure 3:**
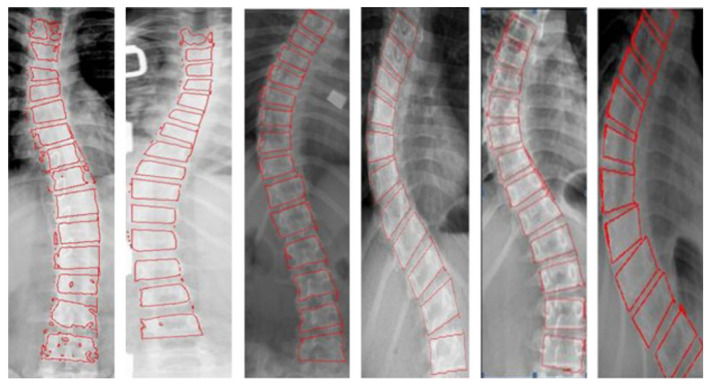
The X-ray images processed by the GVF-snake model [48].

Using filters may preserve some interference features, such as some of the images of the rib with high grayscale. Some researchers have proposed using the active shape model (ASM) to fit the edges of the vertebral body. Williams, D.J. and Shah, M. [47] proposed an active contour model, the snake model, and successfully segmented the vertebral body from the image. However, the snake model has higher requirements for the initial setup. If the initial position of the snake deviates too much from the target, there is a possibility that the snake will not be pulled towards the boundary. Anitha, H. et al. [48] used the GVF snake model to search for the edge of the spine. They replaced the external force with a gradient vector flow (GVF) force in the snake model, which can be defined by the equilibrium solution of the vector diffusion equation. GVF force has fewer requirements for the initial position. As a result, the model acts better in terms of edge extraction. Figure 3 shows the result of Anitha H et al.’s [48] processing of the spine images with the GVF-snake model.

Although the GVF-snake model reduces the active shape model’s dependence on the initial setup, the researchers found that, in some areas with blurred boundaries, GVF-snakes are prone to losing features, especially when the vertebral bodies obscure each other. Roberts, M.G. et al. [49] proposed an active apparent model (AAM) for the identification of spinal vertebral bodies. The active apparent model has two advantages over the active shape model: (1) simple constraints are placed on the parameters, and the resulting shape remains within a reasonable range; (2) a global shape model is used to simulate the correlation between different parts implicitly. Therefore, even if some of the details are occluded, the approximate shape of the occluded part can be estimated by AAM. However, the main idea of its search method is based on the assumption of a linear relationship between the difference between the texture of the model and the area enclosed by the shape and the change in the parameters. This assumption is only reliable within a certain range of bias, and its coverage is highly dependent on the training set. Therefore, the robustness of this method is limited when searching.

In scoliosis cases, the vertebral body may have concomitant aberrations, but neither the active shape model nor the active apparent model can handle the topological changes in the underlying shape. Jalba, A.C. and Wilkinson, M. et al. [50] proposed a shape deformation method based on particle systems: the CPM algorithm. The CPM algorithm treats the grayscale image as an electric field, and the amount of negative charge in the electric field is proportional to the image gradient. A large number of positively charged particles that can be moved freely in the system are gradually concentrated at the edges under the influence of the Lorentz force and the Coulomb force. Sardjono, T.A. et al. [51] improved the CPM algorithm by limiting the direction of movement of the particles and using it to look for the left and right edges of the vertebral body. The improved CPM algorithm performs better for the segmentation of the spinal vertebral body than conventional CPM and GVF-snake models. Since the CPM model does not depend on the initial position of the particles, allowing particles to cross a portion of the boundary freely, and the electric field acts over longer distances relative to other energy fields, it can perform better when the underlying shape topology transformation occurs. Another advantage of CPM is that it can adapt to many unknown topologies and complex shapes, such as edges with large curvature and unclosed profiles. As particles are only subject to force and are not constrained by motion and morphology, they show good robustness in the edge capture of unknown shapes and complex shapes.

### 4.2. Machine Learning Methods for 2D Image

#### 4.2.1. Segment the Vertebral Body to Measure the Cobb Angle

While the image enhancement method has successfully measured Cobb angles on some spine images, it requires precise feature engineering. Thus, there are disadvantages, such as its high computational cost and unstable performance on different images. Machine learning methods can construct a classifier to map a given dataset into several given categories. Some researchers use machine learning methods to segment spine images from X-ray images and then calculate the Cobb angle through spine midline fitting or angle regression network.

Kusuma, B.A. [52] uses a Canny filter to extract the edges of the vertebral body and cooperate with K-means clustering to find the centroid of the spine segment to fit the midline of the spine. To avoid interference from unrelated structures such as ribs, Duong, L. [53] delineated a region of interest (ROI) for each vertebral body in the training set (where ROI is defined as an area that includes the corresponding vertebral body but is slightly larger than each vertebral body). Then, a mean model was calculated to estimate the areas of each vertebral body’s probability in the test set. A Soft-Margin SVM classifier was trained to segment the vertebral body image from each ROI. Finally, the program fitted the midline of the spine and calculated the Cobb angle by calculating the centroid of the vertebral body.

The successful application of the mean model to the selected vertebral ROI demonstrated the usefulness of prior knowledge of anatomy for segmenting medical imaging. However, scoliosis has great individual variability, and the reliability of the mean model is doubtful. Alharbi, R.H. et al. [54] trained convolutional neural networks (CNN) with transfer learning to calibrate ROIs for each spinal vertebral body. CNN performs better in terms of generalization capacity than the mean models. However, this method can only mark the approximate position of each vertebra with a frame, and a classifier is required to know the specific position of each vertebra and the rotation direction.

**Figure 4:**
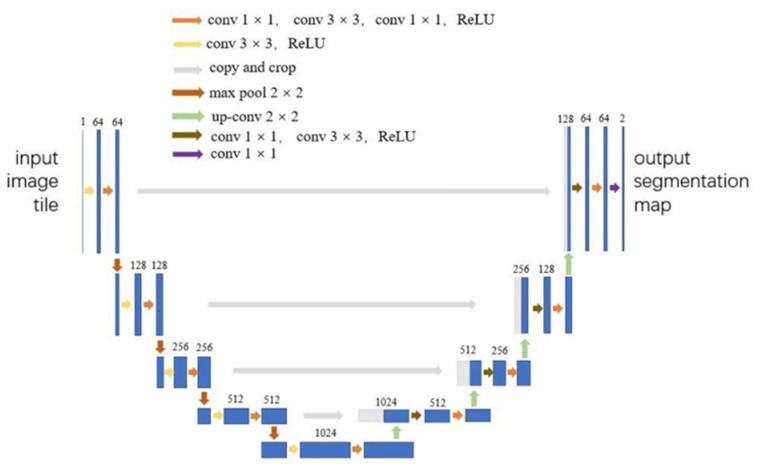
The structure of the improved U-Net [55]: Compared with the original U-net, Wang Z. [58] has made two modifications. First, in order to reduce the amount of calculation, modify conv (3 × 3) to conv (1 × 1) + conv (3 × 3) + conv (1 × 1). In order to reduce the overall parameters, conv (3 × 3) is replaced with conv (1 × 1) when dealing with the same dimension and dimension reduction calculation.

Ronneberger, O. et al. [55] proposed U-Net, which is a semantic segmentation network model based on FCN [56] structure. It is widely used in the field of medical imaging due to its suitability for small datasets and good performance in medical image segmentation. Some researchers have improved the U-net model and used it to measure the Cobb angle. Tu, Y. et al. [57] proposed a DU-net for spine contours’ segmentation. Wang Z. et al. [58] used Resnet to modify U-Net to increase network computing speed while ensuring segmentation accuracy. The structure of the proposed model is shown in Figure 4. The preprocessed image is divided by the improved U-Net into a binary image, where the white area is the vertebral body, and the black area is the non-vertebral body. Then, the minimum external matrix for each spinal vertebral body is calculated, which yields the upper and lower end plates of the vertebral body. Comparing the results of the scoliosis surgeon’s manual method with the improved U-net method, the ICC of both automatic and manual measurement results is higher than 0.96, and the MAD is about 3°. Error analysis also proves good consistency between automatic and manual measurement methods.

Lin, Y. et al. [59] proposed a two-stage learning framework, Seg4Reg. Instead of just using deep learning to segment images, Seg4Reg takes the spine segmentation result as the input of the angle regression network and won first place in the spine image segmentation competition held by MICCAI.

#### 4.2.2. Locate the Landmark of the Spine to Measure the Cobb Angle

We found that, as long as the accurate landmarks of the vertebral body are found, the segmentation can be replaced, and the Cobb angle can be directly estimated. Zhang, C. et al. [60], Chen, B. et al. [61], Kim, K.C. et al. [62], and Zhang, J. et al. [63] have proposed methods to find landmarks of the vertebral body. However, there are also two problems in calculating the Cobb angle by the vertebral landmark: (1) precise feature engineering is required due to image noise; (2) a small error in the landmark may lead to a large deviation of the Cobb angle. Therefore, researchers should not only consider the accuracy of landmark estimation but also consider the impact of reducing landmark errors on Cobb angle measurements.

**Figure 5:**
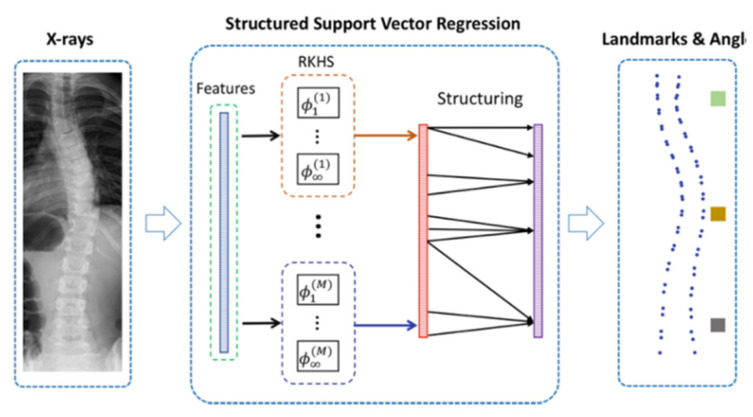
The framework of S^2^VR [64]: SVR is used to capture the angle and landmark output correlation structure matrix, improved to obtain S2VR. Sun, H. et al. [64] introduced manifold regularization and trained with kernel alignment method.

Sun, H. et al. [64] improved the SVR method and proposed a new multi-output regression measurement Cobb angle scheme, S^2^VR. This method introduces manifold regularization into the framework and obtains a joint estimation of Cobb angles and spine landmarks using their high correlation. They compared the Cobb angle estimated by the S^2^VR method with the manually measured Cobb angle, with a Correlation Coefficient (ICC) of 0.923 for the middle angle and 0.884 and 0.902 for the remaining two angles, respectively. The framework of S^2^VR is shown in Figure 5.

**Figure 6:**
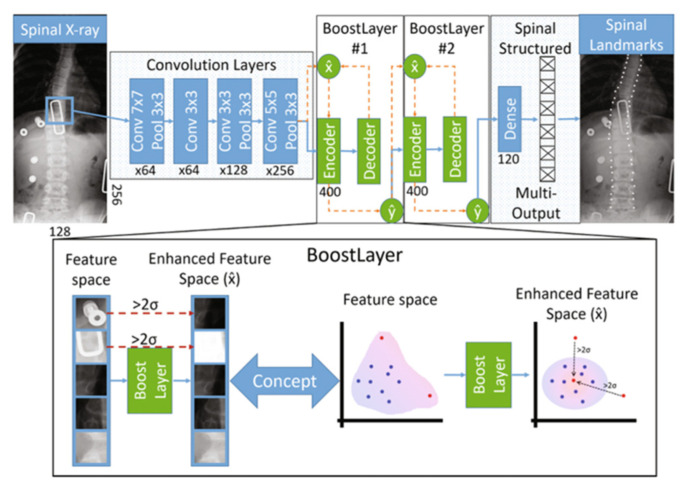
The structure of Boostnet [65]. The Boostnet consists of three parts: a convolutional layer for feature extraction, a boostlayer for removing outliers, and a multi-output layer for relieving the pressure of small datasets by capturing inter-landmark dependencies.

S^2^VR improves the accuracy of Cobb angle and landmark output by considering the explicit dependencies between multiple outputs. However, Wu, H. et al. [65] found that the accuracy of Cobb angle measurements was also affected by outliers in the training data (including subjective error and imaging artifacts). Therefore, they came up with a new framework for automated landmark evaluation: Boostnet. It combines convolutional neural networks with statistical methods. Not only is multi-output regression achieved, but the robustness of landmark estimation is improved. Boostnet uses a dependency matrix of adjacent landmarks to solve the problem of explicit dependencies between multiple outputs. Wu, H. et al. validated Boostnet’s ability to estimate spinal landmarks and found that its mean squared error (MSE) on the training set was only 0.0046. The structure of Boostnet is shown in Figure 6.

**Figure 7:**
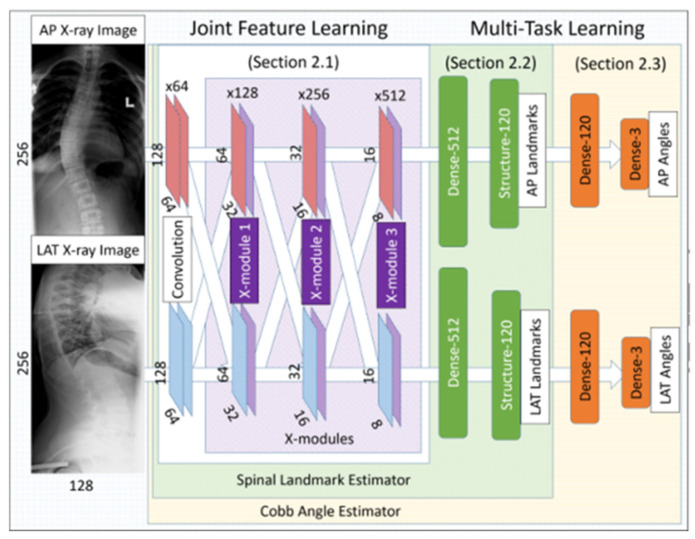
The structure of the MVC-net [67]. MVC-net consists of three parts: a convolutional layer for feature extraction (the convolutional layers are connected with X modules for feature joint learning); a spine landmark output layer and a Cobb angle output layer.

Single-view images tend to miss some features due to the mutual occlusion of vertebral body structures. Wu, H. et al. [66] and Xu Q. [67] noted that multi-view images could complement the missing features of single-view images. Therefore, Wu, H. et al. designed a multi-perspective correlation network (MVC-net) using the X module to perform a weighted learning of feature maps of AP and LAT images to obtain joint features. For the phase-wrapping characteristics between the Cobb angle output and the landmark output, they designed a loss function, considering spinal landmark regression loss, spinal landmark-related loss, and Cobb angle output loss to minimize error. Xu, Q. [67] improved the loss function on the basis of the former and used extrapolation to obtain more accurate angle measurements. The structure of the MVC-net is shown in Figure 7.

Considering the accumulation of errors in the two-stage framework and the fact that the cascaded network cannot guarantee global optimality, Yi Lin et al. [68] proposed Seg4Reg+ by combining the two methods of angle regression and landmark monitoring. Seg4Reg+ trains angle regression and landmark detection methods in parallel and optimizes them globally.

## 5. Evaluation Methods of Cobb Angle in 3D Images

Due to factors such as missing dimensions, measurement noise, and varied postures [69], 2D measurements face great challenges in terms of their accuracy and comprehensiveness. Yang W. et al. [70] measured Cobb angles on three-dimensional reconstructed spinal structures and on X-rays and found that the former has improved accuracy. As 3D imaging technology has become the mainstream means of detection, the methods of 3D Cobb angle measurement using CT, 3D Ultrasonic, or MRI images have gradually become abundant. CT exposes patients to larger doses of radiation [71], which have a negative effect on their health. MRI requires patients to be in a recumbent position, and due to gravity, images of the spine taken in a recumbent position are thought to be very different from those taken in a standing position [72,73,74,75]. Ultrasound imaging is considered especially suitable for adolescent idiopathic scoliosis (AIS) patients for its real-time, cost-effective, and radiation-free characteristics [76]. Although the measurement of 3D images has the characteristics of good continuity and comprehensive observation, due to its complexity for computation, a 3D ultrasound system is still unreliable for clinical scoliosis assessment [77]. Therefore, how to improve the degree of refinement and automation of measurement is also the focus of research by relevant scholars.

### 5.1. Image Enhancement Method for 3D Image

#### 5.1.1. Semi-Automatic Method

Similar to the measurement method used for 2D images, identifying key points through image enhancement and feature detection and then using the prior knowledge of spine shape to fit the curved lines of the spine in a three-dimensional space is the most-used method at present.

Cheung, C.J. et al. [78] developed a freehand 3D ultrasound system for scoliosis curvature measurement. The method used the transverse process and spinous profile as the landmarks. Based on the coronal images, the Cobb angle was measured by volume projection imaging. Vo, Q.N. et al. [79] proposed a method for finding the maximum curvature plane in 3D ultrasound images and using it to measure spinal curvature. The method uses voxel-based reconstruction techniques and bilinear interpolation to reconstruct 3D spine images and then measures the axial rotation of the vertebral body on the 3D images to find the plane of maximal curvature.

#### 5.1.2. Full-Automatic Method

The above methods, which rely on landmark detection, are often used for semi-automated measurements and do not effectively reduce the workload. The full-automatic method generally uses template matching for spinal curve detection.

Volume projection imaging (VPI) is a commonly used method in ultrasound imaging to draw the contours of spiny columns on the coronal surface using the averaging mixing method and to assess scoliosis. Zhou, G. et al. [80] reported an automated method for determining spinal curvature using the ultrasound images reconstructed by VPI. The method identifies spinous tips, using histogram equalization to reduce speckle noise and positioning the darkest points line by line in images after noise suppression. The detected points are then polynomial-fitted to a curve, and the curvature angle of the spine is calculated based on the inflection points on the curve. Polynomial fitting, to some extent, mitigates the effect of misidentified feature points on the measurement results. This method selects the point with the least intensity as the marker. Image intensity is affected by the singularity of the intensity distribution, and when the quality of ultrasound images is low, such as for people whose BMI is high, the accuracy of curve point detection is significantly reduced [81].

In addition, Zhou, G. et al. [82] proposed another method, using prior knowledge of vertebral anatomy to look for features in ultrasound volume projection imaging (VPI). They used a two-fold thresholding strategy to extract the skeletal features, which can obtain information on symmetrical and asymmetric metrics from phase consistency. The spinal contour is detected from the segmented spinal regions and can be used to extract the spinal curve for curvature measurements. However, the recognition accuracy and anti-interference ability of this method are far from satisfactory. In order to solve this problem, Zhou, G. et al. [83] proposed a fully automatic method that facilitates the segmentation of the spinous column profile by adaptively adjusting the frequency bandwidth of the log-Gabor filter to calculate the directional phase consistency.

Visualization techniques such as VPI primarily demonstrate spinal anatomy on the coronal surface, which are inadequately capable of evaluating three-dimensional spinal deformities. Li, D.S. et al. [84] developed a new method based on projection imaging to obtain sagittal images of spinal anatomy. Sagittal projection images and coronal surface VPIs are obtained by deriving spine curves from coronal images at different depths and constructing non-planar surfaces. As long as the sagittal projection image has been calculated, the Cobb angles can be measured automatically.

Some researchers look for markers, such as spinous processes (SP), on images of the spine and fit the three-dimensional curves used to represent the spine. Sagittal projection angle (SPA) is a very common quantitative indicator of three-dimensional spinal curvature. Zeng, H. et al. [85] applied a fully automated method to measure spinous process angle, which was verified to have a high consistency with the Cobb angle, on coronal spinal imagery. This method uses a gradient vector flow (GVF) snake model to locate the spinous process (SP) on ultrasound (US) images of the transverse vertebral body.

Zheng, R. et al. [86,87] proposed an automatic method to measure the Sagittal projection angle (SPA). The method consists of four steps, including spinous process detection, data point clustering, curve fitting, and SPA estimation. Compared with ultrasonic manual measurements, the results of the automated method have a higher correction rate and smaller differences. SPA makes full use of the three-dimensional features of the spine image, but spinal rotation, which is common in scoliosis cases, causes the spinous process to shift more towards the concave side of the spine. Therefore, Herzenberg, J.E. et al. [88] pointed out that the angle of the spinous process may underestimate the severity of curvature of the spine compared to the Cobb method.

For three-dimensional image data, how to improve the image processing speed, considering the image quality and calculation time, is an important problem that needs to be solved. Chen, H.B. et al. [89] used a point projection (FDP) algorithm using voxel-based nearest neighbor (VNN), multiplane interpolation (MPI), and pixel nearest neighbor (PNN) protocols. This method effectively shortens the reconstruction time and can meet the requirements for immediate demonstration while ensuring reliability and accuracy when assessing scoliotic angle measurements. The above methods have a good performance in terms of calculation accuracy and noise suppression. However, in terms of calculation time, 1–2 mins are still needed to process a case, and the efficiency still needs to be further improved.

### 5.2. Machine Learning Method for 3D Image

Scoliosis detection methods based on image characteristics and prior knowledge are susceptible to interference from image noise, resulting in large measurement deviations or even measurement errors. Meanwhile, in medical images, some artificial noise will also be introduced, such as gray features and texture features in US [90] and secondary artifacts in CT. To solve the above problems, researchers have begun to study robust detection methods with noise suppression, mainly using machine learning, deep neural networks, and other frameworks [91,92,93,94].

Considering that a single weak classifier cannot deal with various sources of noise, Wang, C.H. [95] jointly trained multiple weak classifiers through AdaBoost and successfully segmented the spinal cord center points and fitted the spine curve on a CT dataset.

Liu, Z. et al. [96] constructed a fully automatic framework based on Faster R-CNN. The Faster R-CNN is trained to detect vertebral lamina. While the detected lamina pairs are used to fit the midline of the spine curve, a spinal curvature estimator calculates the scoliotic angles based on the curve. This method uses the structural features of the spine, such as ribs and lamina, which are adjacent to each other, as a priori knowledge, but there is still a possibility of false positives in the detection of the spine.

**Figure 8:**
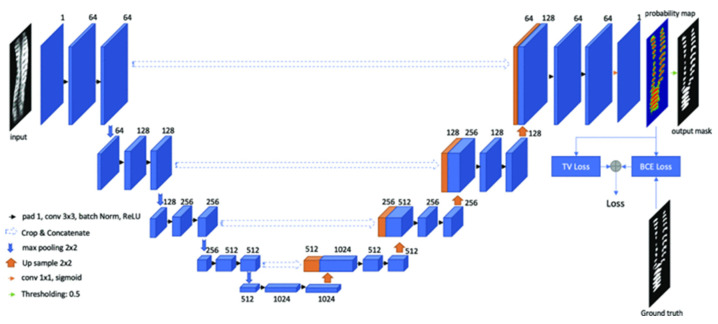
The architecture of RSN-U-net [97]: Similar to U-net, RSN-U-net is also composed of an encoder and decoder. The encoder section consists of four repeated encoder stacks. Each encoder stack contains two conv (3 × 3) convolutional layers and one max-pooling (2). Each decoder stack contains two conv (3 × 3) and one Upsampling (2).

Convolutional neural networks have great advantages in terms of skeletal segmentation and feature extraction, which will facilitate the accurate measurement of scoliosis angles. The most-used method is the U-Net. Huang, Z. et al. [97] introduced a segmentation method called RSN-U-net, as shown in Figure 8. Aiming at the spot and regular occlusion noise that are prone to appear in ultrasound medical images, this method uses total variance (TV) loss to train the neural network and successfully improves the robustness of spot and regular occlusion noise, effectively segmenting the bone features in ultrasound spine images. Agrawal, A. et al. [40] simultaneously used three U-nets to segment the vertebral bodies in the coronal and sagittal planes and the pelvic region of the CT dataset and calculated the sagittal Cobb angle.

Banerjee, S. et al. [98] presented a Light-convolution Dense Selection U-Net (LDS U-Net) to identify lateral bony features from ultrasound spine bony features automatically. In order to export the selective features, the method suppresses irrelevant information with a gating mechanism. In addition, it also uses multi-scale skip-paths to enhance feature fusion.

**Figure 9:**
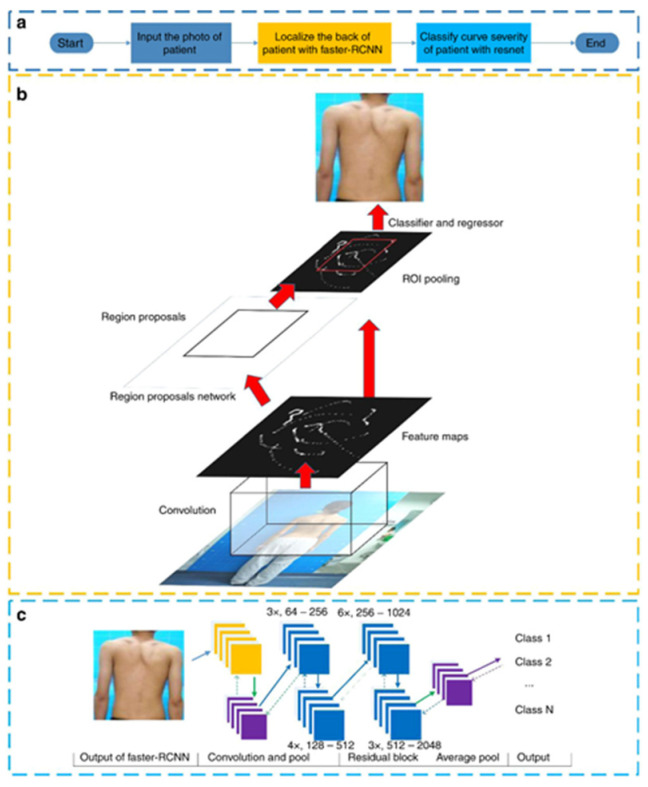
Block diagram and the architectures of Faster-RCNN and Resnet [15]: Yang, J. et al. (**a**) The entire DLA workflow; (**b**) The architecture and workflow of Faster-RCNN; (**c**) The architecture of Resnet. [15]’s algorithm using convolutional layer to obtain a feature map of the back. The algorithm finds the ROI in the feature map, pools it, and classifies it with a classifier.

In addition to the use of 3D medical images, some scholars have used patient back images to directly obtain scoliosis angles through deep learning. This method can avoid radiation damage to the human body and does not require complex medical equipment operation, but the accuracy of its detection and its use in medicine remains to be seen. Yang, J. et al. [15] proposed a deep learning algorithm for automated scoliosis screening using images of uncoated backs, as shown in Figure 9. The algorithm uses Faster-RCNN to locate the back image and classifies the image with Resnet. When performing the initial screening task for scoliosis, the algorithm was both more accurate and efficient than human experts.

Although the experimental results of these methods show that the Cobb angle can be measured effectively, there are also problems, such as calculation time cost and insufficient recognition accuracy, which is also a common problem in two-stage (2-stage) deep learning methods. How to achieve a balance between image quality and the efficiency of computation time is still a major challenge for the current 3D angle measurement algorithms.

## 6. Discussion

With the rapid development of information technology and medical imaging technology, the technical bottleneck of computer-aided measurement of spinal curvature has been continuously broken through. However, the main problems faced by researchers remain challenging.

Due to its low cost and convenience, using 2D medical images for the Cobb angle measurement is always the most important. The greatest problem that researchers encountered at first was the segmentation of the vertebral part of the image. In view of the characteristics of larger noise, blurred edges, and more occlusion between structures, some researchers have used filters, active shape models, and active apparent models to extract the edges of the spine, but the effect is far from satisfactory. The active shape model successfully captures contour features in X-rays but is highly dependent on the initial setup. Furthermore, it fails to solve the problem of overlapping structures in medical images caused by scoliosis, which can lead to the loss of a large number of feature points. The active apparent model has certain constraints on the shape of the object to be segmented, so it performs better in images where structures obscure each other. However, neither the active shape model nor the active apparent model can resolve the underlying changes in topology. While the CPM model can effectively solve the above problem, it is computationally expensive and susceptible to errors caused by variations in X-ray images.

Some researchers have noted that, although there are individual differences in spinal imagery, there are still statistical regularities. Therefore, they use machine learning to segment the spine image and calculate the Cobb angle. One of the most representative methods is Seg4Reg, a two-stage learning framework. However, the stability of the Cobb angle output is still a problem. The output stability of the measurement is affected by a variety of factors, including phase wrapping and vertebral coordinate error. Focusing on the phase wrapping in the angle output, methods such as S2VR, MVC-net, and MVE-net focus on the correlation between the output of the vertebral landmarks and the output of the Cobb angle, with a specific loss function design to ensure a smooth output. Since the appearance of a noise point may affect the selection of the vertebral end and cause a large deviation in the results, researchers proposed the boost-layer method to exclude outliers with large coordinate errors. The AAM model can use statistical methods to estimate the approximate location of feature points. However, it is unable to cope with the fusion and deformation of the vertebral bodies in scoliosis cases.

Machine learning methods and image enhancement methods have their own advantages due to their different technical focus on problem-solving. As Duncan and Ayache [53] pointed out in their review of current medical imaging trends, combining these two areas will help improve accuracy and robustness.

Researchers have identified two drawbacks to using 2D medical images to monitor scoliosis relative to newer technologies: (1) 2D medical images can only show a single perspective, which is prone to information loss caused by occlusion of features; (2) scoliosis is a 3D structural change. The projection on the light image does not reflect the true spine curvature. Some 3D medical imaging technologies, such as MRI and ultrasound, show their advantages, and researchers believe that ultrasound medical imaging is a promising 3D medical imaging for the regular assessment of scoliosis. The information in 3D medical images is often so abundant that the biggest problem facing researchers is how to eliminate interfering information. A problem that troubles researchers is how to reconstruct the 3D structure of the spine. Some researchers have used volume projection imaging (VPI) to describe the spine profile in the coronal plane, but this cannot adequately represent the 3D structural changes in scoliosis. Therefore, some researchers measure the Cobb angle together with VPI by generating a projection of the sagittal plane. Another solution is to reconstruct a curve that describes the trend in the spine by identifying features of the spine, such as spinous or transverse processes, through polynomial fitting. Soon, the researchers encountered two problems. One was that the SPA underestimated the degree of spinal curvature. The researchers realized that we do not have a well-established quantitative assessment of curvature for 3D spinal structures, which is a major source of error in the computer-aided measurement of 3D angles. The other is that the ultrasound images were noisy, and precise feature engineering is required to find the spinous and transverse processes. Therefore, some researchers use filters or introduce machine learning architectures to obtain more robust detection algorithms with noise suppression.

The automatic measurement method for Cobb angle shows its potential application in various medical scenarios, but the existing algorithms are not complete enough. We believe that a complete automatic Cobb angle measurement algorithm should meet five requirements: accurate segmentation and classification, high positioning accuracy, a small angle output error, better robustness for a wider range of application scenarios, and a shorter running time. To achieve this goal, we believe that improvements should be made in the following four areas.

(1) Dataset: The existing automatic measurement algorithms are all data-driven. However, we lack large public datasets for multiple real-world scenarios, and some sources of interference cannot be ignored. For example, intraoperative images have measurement errors caused by image stitching, and there may be interference from surgical instruments. Postoperative imaging may show metallic implants and anatomical abnormalities (e.g., transitional anatomy and missing vertebrae). However, most of the existing databases do not reflect these interference items, which greatly limits their ability to verify the algorithm.

(2) Algorithm design: Current fully automatic algorithms have poor transferability between different datasets, which is reflected in their poor generalization in the face of abnormal anatomy. In the Verse Vertebral Segmentation Competition held by MICCAI in 2019, the vast majority of algorithms performed poorly on the test set, with many anatomical anomalies (such as transitional anatomy and missing vertebral bodies) [99]. Only the algorithm of Payer, C. [100] is stable. In 2020, the situation improved. The algorithm design should not only be based on existing datasets but should also consider the idiotype of the application scenario as much as possible.

(3) Deep learning framework: How to balance image quality and computation time is a big challenge for fully automated Cobb angle measurement methods. It was mentioned earlier that Cobb angle measurements could be used for intraoperative spinal alignment. However, intraoperative automatic measurement requires an extremely high real-time performance, which cannot be solved by the deep learning framework used by existing methods. Therefore, a deep learning framework that considers both efficiency and accuracy is needed.

(4) Application scenarios: Initially, fully automated measurements of Cobb angles were only used for a quantitative assessment of the disease. Now, they can also be used to determine whether the vertebrae are properly aligned during surgery. Future researchers can explore more application scenarios for a fully automated Cobb angle measurement in combination with medical practice. For example, combined with a three-dimensional reconstruction of the spine, the treatment plan, and surgical effect can be evaluated by establishing a mechanical model or combined with real-time registration technology for intraoperative real-time monitoring.

## 7. Conclusions

Measurements of scoliosis angles such as Cobb angles are semi-automatic at present, requiring the manual labeling of marker points to calculate the Cobb angle. These methods are inefficient and inconsistent, and fully automated measurement methods are urgently needed. Research based on 2D images such as X-Ray images is the hotspot and has made great progress; automatic Cobb angle measurements can be achieved in some specific scenarios. For a more comprehensive assessment of scoliosis and lower radioactivity, 3D detection technologies are increasingly used in the assessment of scoliosis, especially adolescent idiopathic scoliosis (AIS). With the improvement in the dataset and the optimization of the deep-learning-based algorithm, automatic measurement of the Cobb angles will be implemented for 3D images, making them applicable to the diagnosis and treatment of spinal curvature.

## Figures and Tables

**Table 1:** Statistical measures for variability of the methods.

Measure	Definition
Technical Error of Measurement (TEM)	$TEM=\frac{\sum_{1}^{N}\left[\sum_{1}^{K}M{\left(n\right)}^{2}-\frac{{\left(\sum_{1}^{K}M\left(n\right)\right)}^{2}}{K}\right]}{N\left(K-1\right)}$
Standard Deviation (SD)	$SD=\sqrt{\frac{1}{N}\overset{N}{\underset{i=1}{\sum }}\left(m_{i}-\bar{m}\right)}$
Average Reliability Coefficient (R)	$R=1-\left[{\left(TEM\right)}^{2}/{\left(SD\right)}^{2}\right]$
Intra- or Inter-class Correlation Co-efficient (ICC)	$ICC=\frac{SD_{\left\{intra\;or\;inter\right\}}^{2}}{SD_{intra}^{2}+SD_{inter}^{2}}$
Mean Absolute Difference (MAD)	$MAD=\frac{1}{N}\overset{N}{\underset{i=1}{\sum }}\left|m_{i}-{\hat{m}}_{i}\right|$
Mean Square Error (MSE)	$MSE=\frac{\sum_{i=1}^{n}{\left(m_{i}-\bar{m}\right)}^{2}}{N}$

## Data Availability

Not applicable.

## References

[1] Li Y.W., Cui W., Yan X.Y., Wang H.J. (2017). Epidemiology of congenital scoliosis in Luohe. Chin. J. Pediatr. Surg..

[2] Wang K., Geng X.M. (2020). Scoliosis is the third biggest killer of children’s health, and the incidence of adolescent girls is eight times higher than that of boys. China Woman’s News.

[3] Moe J.H., Lonstein J.E. (1995). Moe’s Textbook of Scoliosis and Other Spinal Deformities.

[4] Cobb J.T. (1948). Outline for the study of scoliosis. Instructional course lectures. Am. Acad. Orthop. Surg..

[5] Weinstein S.L., Dolan L.A., Cheng J.C., Danielsson A., Morcuende J.A. (2008). Adolescent idiopathic scoliosis. Lacent.

[6] Heary R.F., Albert T.J. (2007). Spinal Deformities.

[7] Aubin C.E., BeUefleur C., Joncas J., de Lanauze D., Kadoury S., Blanke K., Parent S., Labelle H. (2011). Reliability and accuracy analysis of a new semiautomatic radiographic measurement software in adult scoliosis. Spine.

[8] Jeffries B.F., Tarlton M., Desmet A.A., Dwyer S.J., Brower A.C. (1980). Computerized measurement and analysis of scoliosis. Radiology.

[9] Yang W.Z. (2015). Reliability of Cobb Angle Measurements in Severe Congenital Scoliosis. Master’s Thesis.

[10] Shea K.G., Stevens P.M., Nelson M., Masters K.S., Yandow S. (1998). A comparison of manual versus computer-assisted radiographic measurement: Intra-observer measurement variability for Cobb angles. Spine.

[11] Wills B., Auerbach J., Zhu X., Caird M.S., Horn B.D., Flynn J.M., Drummond D.S., Dormans J.P., Ecker M.L. (2007). Comparison of Cobb angle measurement of scoliosis radiographs with preselected end vertebrae: Traditional versus digital acquisition. Spine.

[12] Gstoettner M., Sekyra K., Walochnik N., Winter P., Wachter R., Bach M.C. (2007). Inter- and intra-observer reliability assessment of the Cobb angle: Manual versus digital measurement tools. European Spine Journal.

[13] Stokes I., Aronsson D. (2006). Computer-assisted algorithms improve reliability of King classification and Cobb angle measurement of scoliosis. Spine.

[14] Lin W. (2019). Research of Scoliosis based on Kinect Camera. Master’s Thesis.

[15] Yang J., Zhang K., Fan H., Huang Z., Xiang Y., Yang J., He L., Zhang L., Yang Y., Li R. (2019). Development and validation of deep learning algorithms for scoliosis screening using back images. Commun. Biol..

[16] Liu X., Wang L., Yu M., Liu X.G., Liu Z.J. (2019). DIERS-4D system and QUINTIC gait analysis system for evaluation of spinal- pelvic-lower extremity motor function among different spinal diseases. Orthop. J. China.

[17] Surgimap User Guide For Version 2.3.2 and Higher. https://www.surgimap.com/.

[18] Osher S.J., Rudin L.I. (1990). Feature-Oriented Image enhancement using Shock Filters. SIAM J. Number Anal..

[19] Alvarez L., Mazorra L. (1994). Signal and image restoration using shock filters and anisotropic diffusion. SIAM J. Number Anal..

[20] Gilboa G., Sochen N., Zeevi Y.Y. (2004). Image enhancement and denoising by complex diffusion processes. IEEE Trans. Pattern Anal. Mach. Intell..

[21] Gilboa G., Sochen N., Zeevi Y.Y. (2005). Real and complex PDE-based schemes for image sharpening and enhancement. Adv. Imag. Electron. Phys..

[22] Duong L., Cheriet F., Labelle H. (2010). Automatic Detection of Scoliotic Curves in Posteroanterior Radiographs. IEEE Trans. Bio-Med. Eng..

[23] Wang L., Traub J., Weidert S., Heining S.M., Euler E., Navab N. (2010). Parallax-free intra-operative X-ray image stitching. Med. Image Anal..

[24] Yaniv Z., Joskowicz L. (2004). Long bone panoramas from fluoroscopic X-ray images. IEEE Trans. Med. Imaging.

[25] Wang L., Traub J., Weidert S., Heining S.M., Euler E., Navab N. Parallax-free long bone X-ray image stitching. Proceedings of the International Conference on Medical Image Computing and Computer-Assisted Intervention (MICCAI).

[26] Peleg S., Rousso B., Rav-Acha A., Zomet A. (2000). Mosaicing on adaptive manifolds. IEEE Trans. Pattern Anal. Mach. Intell..

[27] Seamless Stitching Using Multi-Perspective Plane Sweep. https://www.microsoft.com/enus/research/publication/seamless-stitching-using-multi-perspective-plane-sweep/?from=http%3A%2F%2Fresearch.microsoft.com%2Fpubs%2F70064%2Ftr-2004-48.pdf.

[28] Image Alignment and Stitching: A Tutorial. https://www.microsoft.com/en-us/research/publication/image-alignment-and-stitching-a-tutorial/.

[29] Ellingson A.M., Boelter K., Sembrano J.N., Takahashi T., Polly D.W. (2022). Intraoperative stitched fluoroscopic images: Effect of parallax on angular measurements of the spine. Spine J..

[30] Ladd B., Jones K., Polly D. (2021). 2-Dimensional Long Film O-Arm Imaging, an Alternative When Intraoperative Fluoroscopy Is Inadequate. World Neurosurg..

[31] Yuan Y., Gao J.M., Guo L., Cao G. (2015). Ultrasound in Local Soft Tissue Lesions After Orthopaedic Implant Surgery. Chin. J. Med. Imagine.

[32] Zhao S., Robeltson D.D., Wang G., Whiting B., Bae K.T. (2000). X-ray CT metal artifact reduction using wavelets: An application for imaging total hip prostheses. IEEE Trans. Med. Imag..

[33] Meyer E., Raupach R., Lell M., Schmidt B., Kachelrieß M. (2000). Normalized metal artifact reduction (NMAR) in computed tomography. Med. Phys..

[34] Wang G., Snyder D.L., O’Sullivan J.A., Vannier M.W. (1996). Iterative deblurring for CT metal artifact reduction. IEEE Trans. Med. Imaging.

[35] Man B.D., Nuyts J., Dupont P., Marchal G., Suetens P. (2001). An iterative maximum-likelihood polychromatic algorithm for CT. IEEE Trans. Med. Imaging.

[36] Liao H., Lin W.A., Zhou S.K., Luo J.B. (2020). ADN: Artifact disentanglement network for unsupervised metal artifact reduction. IEEE Trans. Med. Imaging.

[37] Lee J., Gu J., Ye J.C. (2021). Unsupervised CT Metal Artifact Learning Using Attention-Guided β-CycleGAN. IEEE Trans. Med. Imaging.

[38] Liao W., Lin H., Peng C., Sun X.H., Zhang J.D., Luo J.B., Chellappa R., Zhou S.H. DuDoNet: Dual Domain Network for CT Metal Artifact Reduction. Proceedings of the IEEE/CVF Conference on Computer Vision and Pattern Recognition (CVPR).

[39] Liao H., Lin W., Huo Z., Vogelsang L., Sehnert W.J., Zhou S.H., Luo J.B. Generative Mask Pyramid Network for CT/CBCT Metal Artifact Reduction with Joint Projection-Sinogram Correction. Proceedings of the International Conference on Medical Image Computing and Computer Assisted Intervention (MICCAI).

[40] Agrawal A., Hietanen A., Särkkä S. Metal Artifact Reduction in Cone-beam Extremity Images Using Gated Convolutions. Proceedings of the International Symposium on Biomedical Imaging (ISBI).

[41] What Is the Radiation Dose for Common Radiological Examinations?. https://www.chunyuyisheng.com/pc/topic/407442/.

[42] Chockalingam N., Dangerfield P.H., Giakas G., Cochrane T., Dorgan J.C. (2002). Computer-assisted Cobb measurement of scoliosis. Eur. Spine J..

[43] Samuvel B., Thomas V., Mini M.G., Renjith Kumar J. A Mask Based Segmentation Algorithm for Automatic Measurement of Cobb Angle from Scoliosis X-Ray Image. Proceedings of the International Conference on Advances in Computing and Communications (ICACC).

[44] Yang D., Lee T., Lai K., Lam T.P., Chu W., Castelein R.M., Cheng J., Zheng Y.P. (2022). Semi-automatic ultrasound curve angle measurement for adolescent idiopathic scoliosis. Spine Deform..

[45] Zhang J.H., Lou E., Hill D.L., Raso J.V., Wang Y.Y., Le L.H., Shi X.L. (2010). Computer-aided assessment of scoliosis on posteroanterior radiographs. Med. Biol. Eng. Comput..

[46] Anitha H., Karunakar A.K., Dinesh K.V.N. (2014). Automatic Extraction of Vertebral Endplates from Scoliotic Radiographs Using Customized Filter. Biomed. Eng. Lett..

[47] Williams D.J., Shah M. (1992). A Fast Algorithm for Active Contours and Curvature Estimation. CVGIP Image Underst..

[48] Anitha H.G., Prabhu K. (2012). Automatic Quantification of Spinal Curvature in Scoliotic Radiograph using Image. J. Med. Syst..

[49] Roberts M.G., Cootes T.F., Adams J.E. Linking Sequences of Active Appearance Sub-Models via Constraints: An Application in Automated Vertebral Morphometry. Proceedings of the British Machine Vision Conference (BMVC).

[50] Jalba A.C., Wilkinson M., Roerdink J. (2004). CPM: A Deformable Model for Shape Recovery and Segmentation Based on Charged Particles. IEEE Trans. Pattern Anal. Mach. Intell..

[51] Sardjono T.A., Wilkinson M., Veldhuizen A.G. (2013). Automatic Cobb Angle Determination From Radiographic Images. Spine.

[52] Kusuma B.A. Determination of spinal curvature from scoliosis X-ray images using K-means and curve fitting for early detection of scoliosis disease. Proceedings of the Information Systems and Electrical Engineering (ICITISEE).

[53] Duncan J.S., Ayache N. (2000). Medical image analysis: Progress over two decades and the challenges ahead. IEEE Trans. Pattern Anal. Mach. Intell..

[54] Alharbi R.H., Alshaye M.B., Alkanhal M.M., Alharbi N.M., Alrehaili O.A. Deep Learning Based Algorithm For Automatic Scoliosis Angle Measurement. Proceedings of the International Conference on Medical Image Computing and Computer-Assisted Intervention (ICCAIS).

[55] Ronneberger O., Fischer P., Brox T. U-net: Convolutional networks for biomedical image segmentation. Proceedings of the International Conference on Medical Image Computing and Computer-Assisted Intervention.

[56] Long J., Shelhamer E., Darrell T. (2014). Fully Convolutional Networks for Semantic Segmentation. IEEE Trans. Pattern Anal. Mach. Intell..

[57] Tu Y., Wang N., Tong F., Chen H. (2019). Automatic measurement algorithm of scoliosis Cobb angle based on deep learning. J. Phys. Conf. Ser..

[58] Wang Z.M., Qin J.Y., Li X., He Z.H. Automatic Image Segmentation and Cobb Measurement of Spine Base on U-Net. Proceedings of the International Automatic Control Conference (CACS).

[59] Lin Y., Zhou H.Y., Ma K., Yang X., Zheng Y.F., Cai Y., Wang L. (2020). Seg4Reg Networks for Automated Spinal Curvature Estimation. Computational Methods and Clinical Applications for Spine Imaging. CSI 2019. Lecture Notes in Computer Science.

[60] Zhang C., Wang J., He J., Gao P., Xie G.T. (2021). Automated vertebral landmarks and spinal curvature estimation using non-directional part affinity fields. Neurocomputing.

[61] Chen B., Xu Q.H., Wang L.S., Leung S., Chung J., Li S. (2019). An Automated and Accurate Spine Curve Analysis System. IEEE Access.

[62] Kim K.C., Yun H.S., Kim S.J., Seo J.K. (2020). Automation of Spine Curve Assessment in Frontal Radiographs Using Deep Learning of Vertebral-Tilt Vector. IEEE Access.

[63] Zhang J., Li H., Ly L., Zhang Y.F. (2017). Computer-Aided Cobb Measurement Based on Automatic Detection of Vertebral Slopes Using Deep Neural Network. Int. J. Biomed. Imaging.

[64] Sun H.L., Zhen X.T., Bailey C., Rasoulinejad P., Yin Y.L., Li S. Direct Estimation of Spinal Cobb Angles by Structured Multi-Output Regression. Proceedings of the International Conference on Information Processing in Medical Imaging.

[65] Wu H., Bailey C., Rasoulinejad P., Li S. Automatic Landmark Estimation for Adolescent Idiopathic Scoliosis Assessment Using BoostNet. Proceedings of the International Conference on Medical Image Computing and Computer-Assisted Intervention.

[66] Wu H., Bailey C., Rasoulinejad P., Li S. (2018). Automated Comprehensive Adolescent Idiopathic Scoliosis Assessment using MVC-Net. Med. Image Anal..

[67] Xu Q. (2019). Accurate Automated Cobb Angles Estimation using Multi-View Extrapolation Net. Master’s Thesis.

[68] Lin Y., Liu L.Y., Ma K., Zheng Y.F. Seg4Reg+: Consistency Learning Between Spine Segmentation and Cobb Angle Regression. Proceedings of the International Conference on Medical Image Computing and Computer-Assisted Intervention.

[69] Vrtovec T., Pernus F., Likar B. (2009). A review of methods for quantitative evaluation of spinal curvature. Eur. Spine J..

[70] Yang W.Z., Li T., Li F., Liu M., Duan C.G., Tao H.R., Luo Z.J. (2015). The Value of Three—Dimensional CT Reconstruction Imaging in Measuring the Cobb Angle in Severe Congenital Scoliosis. Prog. Mod. Biomed..

[71] Karpiel I., Ziębiński A., Kluszczyński M., Feige D. (2021). A Survey of Methods and Technologies Used for Diagnosis of Scoliosis. Sensors.

[72] Morrissy R.T., Goldsmith G.S., Hall E.C., Kehl D., Cowie G.H. (1990). Measurement of the Cobb angle on radiographs of patients who have scoliosis: Evalution of intrinsic error. J. Bone Joint Surg. Am. Vol..

[73] Yazici M., Acaroglu E.R., Alanay A., Deviren V., Cila A., Surat A. (2001). Measurement of vertebral rotation in standing versussupine position in adolescent idiopathic scoliosis. J. Pediatric Orthopaed..

[74] Torell G., Nachemson A., Haderspeck-Grib K., Schultz A. (1985). Standing and supine Cobb measures in girls with idiopathic scoliosis. Spine.

[75] Pandey P.U., Quader N., Guy P., Garbi R., Hodgson A.J. (2020). Ultrasound Bone Segmentation: A Scoping Review of Techniques and Validation Practices. Ultrasound Med. Biol..

[76] Lyn J., Ling S.H., Banerjee S., Zheng J.Y., Lai K.L., Yang D., Zheng Y.P., Bi X.J., Su S., Chamoli U. (2021). Ultrasound volume projection image quality selection by ranking from convolutional RankNet. Comput. Med. Imaging Graph..

[77] Zheng Y.P., Lee T., Lai K., Yip B., Zhou G.Q., Jiang W.W., Cheung J., Wong M.S., Ng B., Cheng J. (2016). A reliability and validity study for Scolioscan: A radiation-free scoliosis assessment system using 3D ultrasound imaging. Scoliosis Spinal Disord..

[78] Cheung C.J., Zhou G.Q., Law S.Y., Mak T.M., Lai K.L., Zheng Y.P. (2015). Ultrasound Volume Projection Imaging for Assessment of Scoliosis. IEEE Trans. Med. Imaging.

[79] Vo Q.N., Le L.H., Lou E. (2019). A semi-automatic 3D ultrasound reconstruction method to assess the true severity of adolescent idiopathic scoliosis. Med. Biol. Eng. Comput..

[80] Zhou G., Zheng Y. Assessment of scoliosis using 3-D ultrasound volume projection imaging with automatic spine curvature detection. Proceedings of the IEEE International Ultrasonics Symposium (IUS).

[81] Cheung C.W., Zhou G.Q., Law S.Y., Lai K.L., Jiang W.W., Zheng Y.P. (2015). Freehand three-dimensional ultrasound system for assessment of scoliosis. J. Orthopaedic Transl..

[82] Zhou G., Jiang W., Lai K., Zheng Y.P. (2017). Automatic Measurement of Spine Curvature on 3-D Ultrasound Volume Projection Image With Phase Features. IEEE Trans. Med. Imaging.

[83] Zhou G.Q., Li D.S., Zhou P., Jiang W.W., Zheng Y.P. (2020). Automating Spine Curvature Measurement in Volumetric Ultrasound via Adaptive Phase Features. Ultrasound Med. Biol..

[84] Li D.S., Zhou G.Q., He Y.K., Zhou P., He S.Y., Zheng Y.P. Ultrasound Sagittal Projection Imaging for the Assessment of Scoliosis. Proceedings of the IEEE International Ultrasonics Symposium (IUS).

[85] Zeng H., Zheng R., Le L.H., Ta D. Measuring Spinous Process Angle on Ultrasound Spine Images using the GVF Segmentation Method. Proceedings of the IEEE International Ultrasonics Symposium (IUS).

[86] Ge S., Zeng H., Zheng R. Automatic Measurement of Spinous Process Angles on Ultrasound Spine Images. Proceedings of the 42 nd Annual International Conference of the IEEE Engineering in Medicine & Biology Society (EMBC).

[87] Zeng H.Y., Lou E., Ge S.H., Liu Z.C., Zheng R. (2021). Automatic Detection and Measurement of Spinous Process Curve on Clinical Ultrasound Spine Images. IEEE Trans. Ultrason. Ferroelectr..

[88] Herzenberg J.E., Waanders N.A., Closkey R.F., Schultz A.B., Hensinger R.N. (1990). Cobb angle versus spinous process angle in adolescent idiopathic scoliosis: The relationship of the anterior and posterior deformities. Spine.

[89] Chen H.B., Zheng R., Qian L.Y., Liu F.Y., Song S., Zeng H.Y. (2021). Improvement of 3-D Ultrasound Spine Imaging Technique Using Fast Reconstruction Algorithm. IEEE Trans. Ultrason. Ferroelectr. Freq. Control..

[90] Huang Q., Zhang F., Li X. (2018). Machine Learning in Ultrasound Computer-Aided Diagnostic Systems: A Survey. BioMed. Res. Int..

[91] Alsinan A.Z., Patel V.M., Hacihaliloglu I. (2019). Automatic segmentation of bone surfaces from ultrasound using a filter-layer-guided CNN. Int. J. CARS.

[92] Salehi M., Prevost R., Moctezuma J.L., Nasvab N., Wein W. Precise Ultrasound Bone Registration with Learning-Based Segmentation and Speed of Sound Calibration. Proceedings of the International Conference on Medical Image Computing and Computer-Assisted Intervention.

[93] Villa M., Dardenne G., Nasan M., Letissier H., Hamitouche C., Stindel E. (2018). FCN-based approach for the automatic segmentation of bone surfaces in ultrasound images. Int. J. CARS.

[94] Gilboa G., Zeevi Y.Y., Sochen N. (2001). Complex Diffusion Processes for Image Filtering.

[95] Wang C.H., Li Y.Z., Ito W., Shimura K., Abe K. (2009). A machine learning approach to extract spinal column centerline from three-dimensional CT data. Proc. SPIE.

[96] Liu Z., Qian L.Y., Jing W.K., Zhou D.S., He X.M., Lou E., Zheng R. Automatic spinal curvature measurement on ultrasound spine images using Faster R-CNN. Proceedings of the IEEE International Ultrasonics Symposium (IUS).

[97] Huang Z., Wang L.W., Leung F., Banerjee S., Yang D., Lee T., Lyn J., Ling S.H., Zheng Y.P. Bone Feature Segmentation in Ultrasound Spine Image with Robustness to Speckle and Regular Occlusion Noise. Proceedings of the IEEE International Conference on Systems, Man, and Cybernetics (SMC).

[98] Banerjee S., Lyu J., Huang Z., Leung H., Lee T., Yang D., Su S., Zheng Y.P., Ling A.H. (2021). Light-Convolution Dense Selection U-Net (LDS U-Net) for Ultrasound Lateral Bony Feature Segmentation. Appl. Sci..

[99] Sekuboyina A., Husseini M.E., Bayat A., Löffler M., Liebl H., Li H.W., Tetteh G., Kukačka J., Payer C., Štern D. (2021). VerSe: A Vertebrae labelling and segmentation benchmark for multi-detector CT images. Med. Image Anal..

[100] Payer C., Štern D., Bischof H., Urschler M. Coarse to Fine Vertebrae Localization and Segmentationwith SpatialConfiguration-Net and U-Net. Proceedings of the International Joint Conference on Computer Vision, Imaging and Computer Graphics Theory and Applications (VISIGRAPP).

